# Spin Frustration Determines the Stability and Reactivity of Metal–Organic Frameworks with Triangular Iron(III)–Oxo Clusters

**DOI:** 10.1002/anie.202514014

**Published:** 2025-09-09

**Authors:** Patrick Lechner, Gaurab Ganguly, Michael J. Sahre, Georg Kresse, Johannes C. B. Dietschreit, Leticia González

**Affiliations:** ^1^ Institute of Theoretical Chemistry Faculty of Chemistry University of Vienna Währinger Str. 17 Vienna 1090 Austria; ^2^ University of Vienna Faculty of Physics Kolingasse 14‐16 Vienna A‐1090 Austria; ^3^ VASP Software GmbH Berggasse 21/14 Vienna A‐1090 Austria; ^4^ University of Vienna Vienna Doctoral School in Chemistry (DoSChem) Währinger Str. 42 Vienna 1090 Austria

**Keywords:** Broken symmetry DFT, Magnetic coupling, Metal–organic framework, Nitrogen adsorption, Spin frustration

## Abstract

Density functional theory (DFT) is the standard approach for modeling MIL‐101(Fe) and related Fe‐based metal–organic frameworks, typically assuming a ferromagnetic high‐spin configuration. However, this widely adopted approach overlooks a key electronic feature: Spin frustration in the triangular ${\mathrm{Fe}}_{3}(\mu_{3}$‐O) nodes. Using flip‐spin, broken‐symmetry DFT, we identify the true ground state as an antiferromagnetic 2S+1=6 state that standard DFT fails to capture. We demonstrate that neglecting spin frustration in MIL‐101(Fe) leads to structural distortions, incorrect energetics, and misleading predictions of stability and reactivity. By explicitly accounting for spin frustration, we recover the correct structure and rationalize the temperature‐dependent $N_{2}$ and CO binding. Spin frustration enhances $N_{2}$ fixation at room temperature, while its loss upon partial ${\mathrm{Fe}}^{\mathrm{III}}$ reduction suppresses this activity but promotes CO adsorption via π‐backbonding. These findings challenge current computational conventions and highlight spin frustration as a critical electronic feature in these frameworks.

## Graphical abstract

Standard DFT fails to capture the spin‐frustrated ground state of triangular Fe(III) metal–organic frameworks (MOFs), leading to distorted geometries and incorrect binding predictions. Using flip‐spin DFT, we identify the true ground state of MIL‐101(Fe) and show how magnetic frustration governs temperature‐dependent $N_{2}$ and CO binding, underscoring the need to treat spin effects accurately.

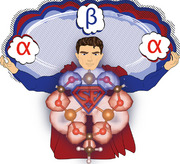

## Introduction

Metal–organic frameworks (MOFs) are revolutionizing the fields of catalysis,^[^ ^1^, ^2^, ^3^ ^]^ spin qubits,^[^ ^4^, ^5^, ^6^, ^7^ ^]^ and gas separation,^[^ ^8^, ^9^ ^]^ owing to their modular architecture, tunable organic linkers, and redox‐active metal centers.^[^ ^10^ ^]^ Among these, iron‐based MOFs with a triangular metal center—particularly MIL‐88B,^[^ ^11^, ^12^ ^]^ MIL‐100,^[^ ^13^ ^]^ and MIL‐101,^[^ ^14^ ^]^ members of the Materials of Institute Lavoisier (MIL) family—have emerged as exceptional catalysts, for example, demonstrating the remarkable ability to catalyze $N_{2}$ fixation^[^ ^15^, ^16^ ^]^ (the conversion of non‐reactive nitrogen gas into ammonia) at ambient conditions,^[^ ^14^, ^17^ ^]^ ${\mathrm{CO}}_{2}$ reduction,^[^ ^18^ ^]^ as well as hydrogen^[^ ^19^ ^]^ and oxygen evolution^[^ ^20^ ^]^ reactions. Despite their impressive catalytic potential, the mechanisms that drive these transformations remain poorly understood, particularly at the atomistic level.

**Figure 1:**
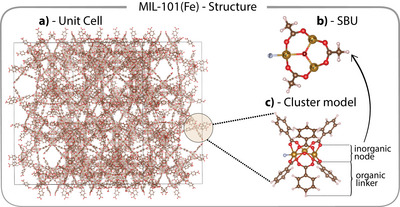
a) Unit cell of the MOF MIL‐101(Fe). b) Top view of the inorganic node of the secondary building unit (SBU), where benzene‐1,4‐dicarboxy (BDC) linkers have been replaced by acetic acid for easier visualization. c) full cluster model for MIL‐101(Fe), in which BDC linkers are replaced with benzoic acid for charge neutrality. The golden circle in panel (a) highlights one cluster to emphasize the relative size of the cluster compared to the entire unit cell.

MIL‐101(Fe) comprises approximately 16 000 atoms within its unit cell (Figure 1a).^[^ ^14^ ^]^ The catalytic activity of MIL‐101(Fe) is governed by its triangular [${\mathrm{Fe}}_{3}^{\text{III}}(\mu_{3}$‐O)] building unit (Figure 1b,c), in which three ${\mathrm{Fe}}^{\mathrm{III}}$ centers—bridged by a central $\mu_{3}$‐oxo atom and connected via benzene‐1,4‐dicarboxylate (BDC) linkers—collectively modulate the material's electronic structure and reactivity. MIL‐101(Fe) exhibits higher $N_{2}$ fixation activity at room temperature compared to its chromium analogue, MIL‐101(Cr).^[^ ^15^ ^]^ However, its reactivity declines sharply at elevated temperatures,^[^ ^15^ ^]^ indicating a thermally sensitive catalytic mechanism that is not well‐understood. Interestingly, CO absorption with MIL‐101(Fe) follows an opposite trend, with higher rates observed at elevated temperatures.^[^ ^21^, ^22^ ^]^

Recent studies have shown that the trinuclear [${\mathrm{Fe}}_{3}^{\text{III}}(\mu_{3}$‐O)] node, when incorporated into a structurally similar MOF (CCDC 1042546), exhibits antiferromagnetic coupling mediated by superexchange through the bridging $\mu_{3}$‐oxo atom.^[^ ^23^ ^]^ Superexchange^[^ ^24^, ^25^, ^26^ ^]^ can be understood as a stabilizing indirect exchange interaction between non‐neighboring magnetic ions (${\mathrm{Fe}}^{\mathrm{III}}$), which is mediated by the non‐magnetic bridging oxygen ($\mu_{3}$‐O).

**Scheme 1:**
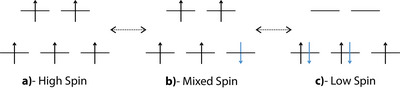
High‐Spin a), Mixed‐Spin b), and Low‐Spin c) configurations of a complex with a single ${\mathrm{Fe}}^{\mathrm{III}}$ center.

In a complex containing a single ${\mathrm{Fe}}^{\mathrm{III}}$ center, multiple spin states are possible. The high‐spin state corresponds to a spin quantum number S=5/2, giving a multiplicity M=2S+1=6, with all five unpaired electrons aligned parallel (Scheme 1a). Mixed spin states with S=3/2 are in principle a possibility, depending on the ligand field strength and electronic configuration (Scheme 1b). The low‐spin state has S=1/2, where only one electron is unpaired, resulting in M=2 (Scheme 1c).

**Figure 2:**
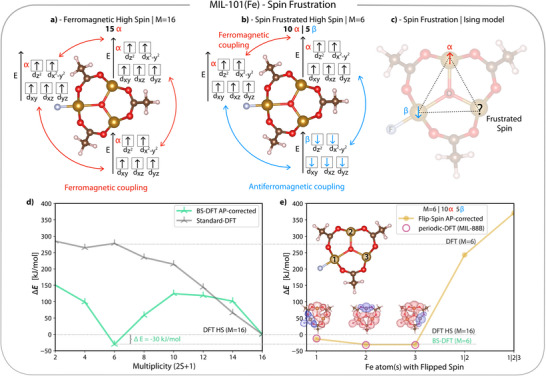
a) Electron configuration of the high‐spin configuration with a total multiplicity (M) of 16. All spins are aligned, leading to ferromagnetic coupling (red arrows) between all three ${\mathrm{Fe}}^{\mathrm{III}}$. b) Spin‐frustrated configuration with a total M=6. Here, 5 spins on one ${\mathrm{Fe}}^{\mathrm{III}}$ are flipped to β down (blue), resulting in antiferromagnetic coupling (blue arrows) with the remaining ten ferromagnetic coupled α up spins localized on the other two ${\mathrm{Fe}}^{\mathrm{III}}$. c) Illustration of spin‐frustration. Inorganic node overlaid with the Ising‐triangle, where the spins on the top ${\mathrm{Fe}}^{\mathrm{III}}$ are α up, while those on the left iron align antiparallel as β down. The remaining ${\mathrm{Fe}}^{\mathrm{III}}$ is frustrated, experiencing equal preference for either spin orientation. d) energies for the spin ladder with respect to the ferromagnetic high‐spin M=16 configuration. Standard unrestricted Kohn‐Sham DFT results for each multiplicity in light gray and broken‐symmetry DFT (BS‐DFT) energies, corrected via the approximate projection (AP) method, in light green. e) Energies relative to ferromagnetic M=16 configuration obtained with the flip‐spin (FS‐DFT) approach. The lower dashed line indicates the BS‐DFT energy corresponding to M=6. The *x*‐axis denotes the ${\mathrm{Fe}}^{\mathrm{III}}$ center(s) on which the spin was flipped (see inlay of inorganic node for numbering of atoms). The spin densities above 1, 2, and 3 show the result of flipping the spin on the corresponding ${\mathrm{Fe}}^{\mathrm{III}}$. Points 1|2 and 1|2|3 are obtained by distributing the flipped spins over two or three ${\mathrm{Fe}}^{\mathrm{III}}$ centers. The dashed line at around 270 kJ/mol indicates the energy of electron configuration M=6 for standard DFT. The purple circles indicating the energies relative to ferromagnetic M=16 configuration obtained with periodic‐DFT on MIL‐88B (no coordinated water).

When several iron centers couple, as in MIL‐101(Fe), a larger number of spin states are possible. In ferromagnetic coupling, the total spin moments on each site align in the same direction (Figure 2a), whereas in perfect antiferromagnetic coupling, the magnetic moments of two sites cancel each other (total M=1), although each center may have a multiplicity greater than one (blue interaction in Figure 2b). An inorganic node with a triangular arrangement of spin centers cannot reach complete antiparallel spin alignment—a phenomenon known as spin frustration, in which competing antiferromagnetic interactions prevent the system from adopting a unique spin ground state. Specifically, two antiparallel‐aligned high‐spin ${\mathrm{Fe}}^{\mathrm{III}}$ centers induce degeneracy in the third, yielding a spin frustrated configuration that is reminiscent of the Ising triangle model (Figure 2c).^[^ ^27^, ^28^, ^29^ ^]^ Since spin frustration affects each spin center, the ground state wave function has a strong multireference character.^[^ ^30^ ^]^ Although spin frustration is well‐known in low‐dimensional magnetic systems, its manifestation in a three‐dimensional MOF such as MIL‐101 is rare^[^ ^23^, ^31^ ^]^ and may hold the key to understanding the material's catalytic reactivity.

Spin frustration has long fascinated condensed matter physicists due to its connection with exotic quantum phenomena, including anomalous quantum Hall effect,^[^ ^32^ ^]^ quantum spin liquids,^[^ ^33^, ^34^, ^35^, ^36^ ^]^ and topologically protected states.^[^ ^37^, ^38^ ^]^ Its observation in a three‐dimensional MOF, such as MIL‐101(Mn),^[^ ^39^ ^]^ is particularly intriguing, and could help to explain the increased catalytic activity of the iron‐based versions of MOFs MIL‐88B, MIL‐100, and MIL‐101 in comparison to MIL‐53(Fe), where the iron centers are linearly aligned.^[^ ^17^ ^]^ The larger pore size of MIL‐101(Fe) and thus larger surface area make it a more accessible catalyst compared to the more densely packed MIL‐88B(Fe), which otherwise consists of identical building units. However, the complexity and scale of the MIL‐101(Fe) and related structures poses significant challenges for high‐level theoretical modeling. As a result, current simulations focus on cluster models representing smaller, chemically relevant fragments of the framework. Conventional Kohn‐Sham density functional theory (DFT) studies, primarily using ferromagnetically coupled high‐spin centers, have become the *de facto* standard approach.^[^ ^3^, ^15^, ^40^, ^41^, ^42^, ^43^, ^44^, ^45^, ^46^ ^]^ For instance, several previous studies^[^ ^47^, ^48^, ^49^ ^]^ have calculated the ground state of MIL‐101(Fe) assuming a fully ferromagnetically coupled configuration, thereby neglecting spin frustration.

Herein, we present a computational framework that explicitly incorporates spin frustration into the electronic structure models of MIL‐101(Fe). Our results demonstrate that conventional ferromagnetic high‐spin‐DFT calculations result in distorted geometries with respect to the crystal structure, which would suggest that MIL‐101(Fe) is unstable in the presence of water in which it is synthesized.^[^ ^14^ ^]^ We show that explicit inclusion of spin frustration in the electronic structure calculations recovers the correct ground‐state geometry and yields a more accurate depiction of MIL‐101(Fe)'s stability and electronic properties. Furthermore, we demonstrate that spin frustration significantly influences the catalytic properties of MIL‐101(Fe), particularly in $N_{2}$‐fixation, thus shedding light on its thermally sensitive reactivity. This work not only challenges existing theoretical models of MIL‐101(Fe), but also opens a new framework for understanding spin‐frustrated systems in three‐dimensional MOFs.

## Results and Discussion

Ye et al.^[^ ^49^ ^]^ reported standard DFT calculations across the full spin ladder of MIL‐101(Fe), scanning over all possible even multiplicities (from M=16 for the ferromagnetically coupled high‐spin state down to M=2 with each iron center in a mixed spin state), and concluded that the fully parallel, high‐spin M=16 configuration (Figure 2a) is the most stable. Each total multiplicity lower than M=16 corresponds to several possible spin arrangements. Using standard (single‐determinant, unrestricted Kohn‐Sham) DFT on the small cluster depicted in Figure 1b, we can confirm these results (gray curve in Figure 2d).

However, standard unrestricted DFT reliably captures only the high‐spin (M=16) ferromagnetic configuration. In contrast, states with lower total spin multiplicities (M=14 to M=2), which exhibit substantial multireference character, lie beyond the scope of standard single‐reference Kohn‐Sham DFT. To access these configurations within a single‐determinant framework, we employ broken‐symmetry DFT (BS‐DFT), which provides an approximate yet practical treatment of antiferromagnetic and intermediate‐spin states.^[^ ^50^, ^51^ ^]^ The success of BS‐DFT depends sensitively on the initial spin‐density guess and typically requires careful preparation of the structural and electronic inputs to ensure reliable convergence.

Following convergence of the high‐spin state, BS‐DFT achieves symmetry breaking by spatially separating α and β spin densities into localized orbitals, allowing singly occupied orbitals to align with opposing spin orientations on different metal sites.^[^ ^50^, ^51^, ^52^ ^]^ For MIL‐101(Fe), the BS solution is constructed by localizing the 15 singly occupied molecular orbitals on the three Fe centers and adjusting the number of β spins according to the desired BS solution. Due to spin‐symmetry breaking, BS‐DFT suffers from significant spin contamination. To estimate the energy of a pure spin state, we apply the approximate spin projection (AP) method introduced by Yamaguchi et al.^[^ ^53^ ^]^ The AP correction method improves spin purity at the energy level, but does not recover the exact spin eigenfunction or fully reestablish spin symmetry in the wavefunction itself. For all calculations, the PBE0‐D3(BJ)/def2‐SVP/def2‐TZVP@Fe /CPCM(water)^[^ ^54^, ^55^, ^56^, ^57^, ^58^ ^]^ level of theory was employed (see further details in Section S1 and a comparison of functionals in Figure S1).

Scanning the entire spin ladder with AP‐corrected BS‐DFT reveals that the state with lower total multiplicity M=6 is energetically favored by 30 kJ/mol over the ferromagnetically coupled, high‐spin configuration (M=16), see light green curve in Figure 2d. Since the broken‐symmetry state with total spin projection M=6 is the only configuration along the spin ladder that is energetically favored over the fully ferromagnetic M=16 state, we focus exclusively on the M=6 and M=16 configurations in all subsequent calculations. BS‐DFT only allows control over the total number of α and β electrons but not their spatial localization; therefore, we employed site‐specific flip‐spin BS‐DFT (or FS‐DFT for brevity) to better characterize the broken‐symmetry M=6 state. FS‐DFT builds on BS‐DFT by enabling targeted α→β spin flips on selected atoms, here the ${\mathrm{Fe}}^{\mathrm{III}}$ centers (Section S1). This approach provides direct access to specific spin states, linking electronic configurations to local magnetic structures and enabling a chemically intuitive understanding of spin‐state energetics and site‐specific magnetism.^[^ ^52^, ^59^, ^60^ ^]^ For instance, achieving a spin multiplicity of M=6 in a system with 15 α electrons distributed across three ${\mathrm{Fe}}^{\mathrm{III}}$ centers requires five α→β spin flips (Figure 2b). These five flipped β spins can be localized on a single ${\mathrm{Fe}}^{\mathrm{III}}$ center or distributed over multiple sites, corresponding to a localized or delocalized spin configuration, respectively. Figure 2e displays the relative energies of these FS‐DFT configurations.

Our results show that the spin‐frustrated, high‐spin state with M=6—where all five flipped beta spins are localized on a single ${\mathrm{Fe}}^{\mathrm{III}}$ center (1, 2, or 3)—is energetically more favorable than the fully ferromagnetic M=16 high‐spin state (see Table S1). In contrast, configurations where the flipped spins are distributed across multiple ${\mathrm{Fe}}^{\mathrm{III}}$ centers (e.g., 1|2 or 1|2|3) lie 250–350 kJ/mol higher in energy than the M=16 state. This indicates that spin localization on a single Fe center, where all ${\mathrm{Fe}}^{\mathrm{III}}$ centers adopt local high‐spin configurations and couple antiferromagnetically where possible, yields the most stable configuration. Conversely, delocalizing the flipped spins induces local mixed‐spin character, which is energetically unfavorable, due to reduced exchange interaction in line with Hund's rule.

These findings agree with those of Mavrandonakis et al.,^[^ ^41^ ^]^ who reported that mixed‐spin configurations at individual ${\mathrm{Fe}}^{\mathrm{III}}$ centers are disfavored by up to 270 kJ/mol. However, their study only considered delocalized broken‐symmetry solutions, where the flipped spins were spread across multiple ${\mathrm{Fe}}^{\mathrm{III}}$ sites. Since these states lie above the M=16 configuration, they concluded that the ferromagnetic state represents the true ground state. In contrast, our calculations reveal a hitherto unreported *localized* M=6 spin‐frustrated state that lies significantly lower in energy than both the *delocalized* M=6 and the ferromagnetic high‐spin M=16 configurations.

Spin‐density isosurfaces (Figure 2e) provide direct evidence for this localization, showing a sharply concentrated β spin density at Fe centers 1, 2, or 3. The nearly identical energetic stabilization across these three centers suggests a degenerate multireference ground state. Overall, our results demonstrate that spin‐frustration is intrinsic to MIL‐101(Fe), consistent with the experimentally observed antiferromagnetic coupling in a structurally related MOF (CCDC 1042546)^[^ ^23^ ^]^ and several non‐periodic [${\mathrm{Fe}}_{3}^{\text{III}}(\mu_{3}$‐O)] complexes.^[^ ^61^, ^62^, ^63^, ^64^ ^]^

To assess whether the local spin‐frustration observed in the cluster model of MIL‐101(Fe) persists in the extended lattice, we also performed periodic DFT calculations. Due to the prohibitively large unit cell of MIL‐101—containing over 16 000 atoms and rendering fully periodic plane‐wave calculations computationally very demanding—we instead investigated MIL‐88B(Fe). This topologically related MOF preserves the key [${\mathrm{Fe}}_{3}^{\text{III}}(\mu_{3}$‐O)] node bridged by BDC linkers, but features a much smaller unit cell containing only two triangular Fe nodes and 120 atoms in total (Sections S2 and S3). Importantly, the periodic plane‐wave calculations with VASP^[^ ^65^ ^]^ (see purple circles in Figure 2e) yield the same energy ordering of the spin states as the cluster model. The states in which the β‐electrons are localized on one of the undercoordinated Fe‐ions (Fe‐2 and Fe‐3 in Figure 2) are 32 kJ/mol below the ferromagnetic state in the periodic calculations. The state with the β‐electrons localized at the Fe coordinated by $F^{-}$ (Fe‐1) is 12  kJ/mol lower in energy than the ferromagnetic state. These results are in excellent quantitative agreement with those of the cluster model, where the spin frustrated states are stabilized by 27 and 12 kJ/mol relative to the ferromagnetic state, respectively (Figure 2e). We note that the reported energy differences for the periodic calculations are not corrected for spin contamination since the necessary quantity is currently not implemented in VASP. However, this does not affect our conclusions, because the AP energy corrections for the cluster model are only around 2–3 kJ/mol.

Furthermore, we wanted to study the potential interactions between the different [${\mathrm{Fe}}_{3}^{\text{III}}(\mu_{3}$‐O)] clusters in the extended system, particularly whether they affect the spin frustration observed for the single cluster. The MIL‐88B(Fe) unit cell contains two [${\mathrm{Fe}}_{3}^{\text{III}}(\mu_{3}$‐O)] clusters. To probe possible interactions between them, we localized in one simulation the β‐electrons on Fe‐1 in one cluster (coordinated by $F^{-}$) and on an undercoordinated iron ion (Fe‐2 or Fe‐3) in the other cluster. The energy of the unit cell in this system was the average of the energies of a unit cell in which the β‐electrons were localized at Fe‐1 in both Fe‐clusters and the unit cell in which the β‐electrons were localized at Fe‐2 or 3 in both clusters (Figure S8). Furthermore, we changed the position of the fluoride in one of the two clusters, as shown in Figure S7B, and calculated the energy of this configuration with both clusters in either the ferromagnetic M=16 or the spin‐frustrated M=6 configuration. The energy difference for the asymmetric fluoride distribution (31 kJ/mol) was within 1 kJ/mol of the one shown in Figure S7A (32 kJ/mol). These findings strongly suggest that in the extended system the Fe(III)‐oxo clusters do not interact. This is in line with the quantitative agreement obtained between the single‐cluster model and the periodic system regarding the energetic ordering of the different spin states. Collectively, these results point to the fact that the spin‐frustrated state is the true ground state of the system and that a single‐cluster model is sufficient to describe the electronic properties of an Fe(III)‐oxo center in the extended framework.

**Scheme 2:**
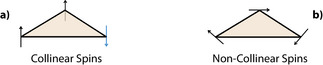
Collinear versus non‐collinear spins.

Since non‐collinear spin arrangements can be important when describing open‐shell states,^[^ ^66^ ^]^ we also explicitly investigated whether the collinear solution is stable with respect to non‐collinear spin relaxation. Actually, spin‐frustration (Scheme 2a) can be further reduced if the spins are canted to form 120 angles with each other, as depicted in Scheme 2b, with total multiplicity M=1.^[^ ^67^, ^68^ ^]^ Accordingly, we performed periodic non‐collinear plane‐wave DFT calculations^[^ ^65^, ^69^ ^]^ on the MIL‐88B(Fe) to investigate whether this canted spin arrangement is more stable than the spin‐frustrated one. After undergoing electronic relaxation, the system adopts a configuration in which two of the ${\mathrm{Fe}}^{\mathrm{III}}$ centers are antiferromagnetically coupled and the third ${\mathrm{Fe}}^{\mathrm{III}}$ center has a spin rotated 90 with respect to the other two ${\mathrm{Fe}}^{\mathrm{III}}$ centers (Figure S9B). However, the configuration shown in Scheme 2a is only slightly higher in energy (0.2 kJ ${\mathrm{mol}}^{-1}$ per Fe‐cluster, see Figure S9D), making it an excellent proxy for the lower energy non‐collinear structure. The preference for the lowest energy spin arrangement can be rationalized by the asymmetry of the [${\mathrm{Fe}}_{3}^{\text{III}}(\mu_{3}$‐O)] cluster. The ${\mathrm{Fe}}^{\text{III}}$ ion coordinated to a negatively charged ligand (Fe‐1) exhibits a longer Fe‐$O_{\mu_{3}}$ bond (2.11 Å) than the remaining two Fe─$O_{\mu_{3}}$ bonds, which are decidedly shorter (1.81 Å). The shorter Fe─$O_{\mu_{3}}$ bonds enhance the superexchange interaction between the two ${\mathrm{Fe}}^{\mathrm{III}}$ centers (Fe‐2 and Fe‐3), mediated via the bridging $\mu_{3}$‐O. To maximize the superexchange, the spins at Fe‐2 and Fe‐3 should be antiparallel. The Fe‐1 center now remains in a spin‐frustrated state, leading to a 90 canted non‐collinear ground state structure. However, aligning the spin on the Fe‐1 atom with any of the other two Fe spins leads only to a slight increase of the energy. This indicates a weak superexchange coupling of the Fe‐1 center to the remaining two Fe centers. Disregarding the slight preference for the non‐collinear solution, a single cluster model reliably captures the key electronic structure features of the extended MIL‐101(Fe) framework.

**Figure 3:**
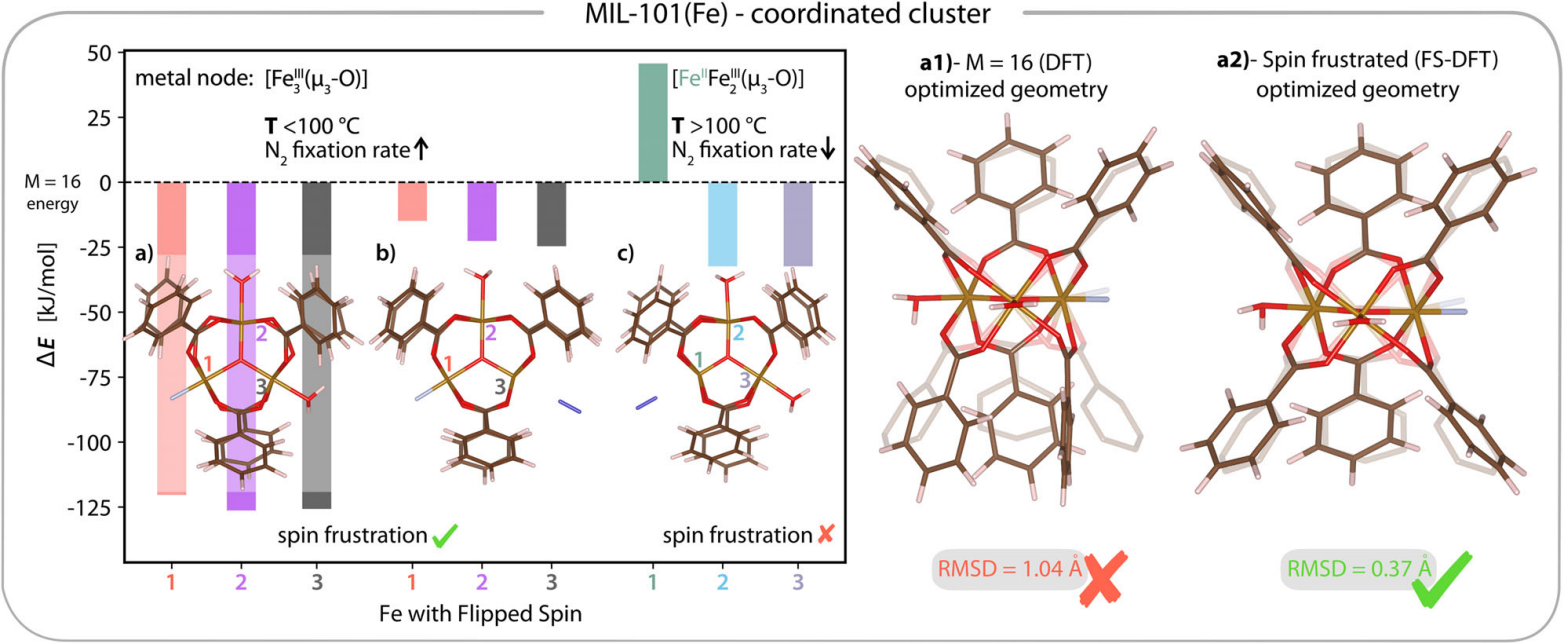
a–c) Energy difference (ΔE) between FS‐DFT calculated clusters and the ferromagnetic high‐spin (M=16) reference. Each bar represents the energy of flipping the spin to β down on the corresponding ${\mathrm{Fe}}^{\text{III}}$ or ${\mathrm{Fe}}^{\text{II}}$. a) Cluster containing a [${\mathrm{Fe}}_{3}^{\text{III}}(\mu_{3}$‐O)] node coordinated with two $H_{2}O$. b) Cluster containing a [${\mathrm{Fe}}_{3}^{\text{III}}(\mu_{3}$‐O)] node coordinated with $H_{2}O$ and $N_{2}$. c) Cluster containing a [${\mathrm{Fe}}^{\text{II}}$ ${\mathrm{Fe}}_{2}^{\text{III}}(\mu_{3}$‐O)] node coordinated with two $H_{2}O$ and $N_{2}$. a1,a2) structural comparison of the water coordinated cluster optimized for, a1) the ferromagnetic high spin M=16 (DFT), and a2), the spin‐frustrated (calculated with FS‐DFT) case, demonstrating that the M=16 configuration results in a distorted structure. The experimental crystal structure is presented transparent behind each structure, to visualize distortion. The RMSD is presented in Å  for each cluster with respect to the crystal structure.

Based on this validation, we further use the cluster model to investigate $N_{2}$ and CO binding. MIL‐101(Fe) has been synthesized and exhibits $N_{2}$ fixation activity in aqueous solution,^[^ ^14^, ^15^ ^]^ necessitating the explicit inclusion of $H_{2}O$ molecules to accurately capture the local coordination environment during $N_{2}$ fixation. Accordingly, our cluster model incorporates the charge neutral benzoic acid linkers and coordinated $H_{2}O$ molecules (Figure 3) to reflect realistic conditions. Even if ${\mathrm{NH}}_{3}$ production requires light activation, we propose that $N_{2}$ coordination can occur already in the dark.

Our FS‐DFT calculations reveal that the spin‐frustrated configuration of the cluster model with two additional coordinated waters (Figure 3a) is dramatically favored by approximately 120  kJ/mol with respect to the ferromagnetic high‐spin configuration (M=16). Notably, optimizing the cluster model for MIL‐101(Fe) with two $H_{2}O$ ligands coordinated to the remaining two ${\mathrm{Fe}}^{\mathrm{III}}$ centers in the ferromagnetic high‐spin configuration (M=16) yields a significantly distorted geometry. Accordingly, the resulting optimized structure (Figure 3a1) deviates from the experimental crystal structure with a root‐mean‐square deviation (RMSD) of 1.04 Å (excluding hydrogen atoms and the two coordinating $H_{2}O$ molecules). This is particularly concerning, as MIL‐101(Fe) inherently contains coordinated waters due to its synthesis in aqueous solution.^[^ ^14^ ^]^ Yet, standard ferromagnetic DFT (M=16) incorrectly predicts MIL‐101(Fe) to be geometrically unstable under such experimental conditions. In contrast, the FS‐DFT optimization in a spin‐frustrated configuration (M=6) yields a geometry with an RMSD of only 0.37 Å (Figure 3a2)—in excellent agreement with the crystal structure and consistent with its experimental stability. Thus, we are left to conclude that the distortions shown in Figure 3a1 are a direct consequence of incorrect modeling MIL‐101(Fe) in the ferromagnetic high‐spin state. These findings highlight the critical role of spin frustration in accurately modeling triangular ${\mathrm{Fe}}^{\text{III}}$ MOFs. Ye et al.^[^ ^49^ ^]^ reported optimized structures of the same cluster model containing benzoic acid linkers, fluoride, and two coordinated $H_{2}O$, based on time‐dependent DFT (TD‐DFT) in the ferromagnetic high‐spin configuration. The similarity between their reported geometry (in Figure 5 of reference [49]) and our distorted high‐spin structure (Figure 3a1) indicates that the standard ferromagnetic high‐spin approach not only yields erroneous ground‐state results, but also affects the reliability of excited‐state properties predicted by TD‐DFT starting from the M=16 reference.

**Figure 4:**
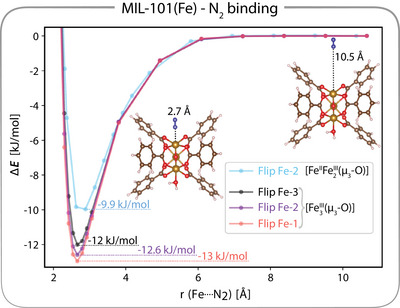
Energy profiles for $N_{2}$ binding, with unbound geometry (Fe‐$N_{2}$ bond distance of 10.5 Å) set to 0 kJ/mol. Each curve (orange, violett and black) corresponds to a configuration with spin flipped to β on a different ${\mathrm{Fe}}^{\mathrm{III}}$ center for the [${\mathrm{Fe}}_{3}^{\text{III}}(\mu_{3}$‐O)] configuration. The blue curve represent flipping the spin of the water coordinated ${\mathrm{Fe}}^{\mathrm{III}}$ for the [${\mathrm{Fe}}^{\text{II}}$ ${\mathrm{Fe}}_{2}^{\text{III}}(\mu_{3}$‐O)] configuration. The energy gain upon $N_{2}$ coordination are given explicitly for each flip‐spin configuration.

When the complex is coordinated with both $H_{2}O$ and $N_{2}$ (Figure 3b), the spin‐frustrated configuration is also predicted to be most favorable, with an energy difference to the high‐spin configuration (M=16) close to that obtained in the pristine cluster (cf. Figure 2d,e). Li et al.^[^ ^15^ ^]^ experimentally observed a strong decrease in the ability of MIL‐101(Fe) to bind nitrogen at high temperatures (T>100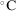). Their ferromagnetic high‐spin DFT calculations could not explain this behavior.^[^ ^15^ ^]^ To shed light on the matter, we calculated the energy profiles for the $N_{2}$‐binding by performing relaxed geometry scans in the flip‐spin approach, starting from the FS‐DFT optimized structure and elongating or shortening the Fe─$N_{2}$ bond for all three flip‐spin configurations (i.e., allowing each Fe center to carry the β spins that results in M=6). Figure 4 shows that $N_{2}$ binds end‐on to the MIL‐101(Fe) framework with a stabilization energy of 12–13  kJ ${\mathrm{mol}}^{-1}$ depending on the localization of the β spins. In contrast, geometry scans using the ferromagnetic high‐spin (M=16) configuration converged, but the cluster remained stable only at the optimized Fe─$N_{2}$ bond length, while any deviation led to structural distortions similar to those depicted in Figure 3a1 (see Figure S3 for a detailed comparison).

Upon heating, the framework decreases its fluoride content from an average 0.81 to 0.58 $F^{-}$ per inorganic node corresponding to a reduction of one of the ${\mathrm{Fe}}^{\mathrm{III}}$ centers to ${\mathrm{Fe}}^{\text{II}}$ ([${\mathrm{Fe}}^{\text{II}}$ ${\mathrm{Fe}}_{2}^{\text{III}}(\mu_{3}$‐O)]), while the other two remain in the ${\mathrm{Fe}}^{\mathrm{III}}$ oxidation state^[^ ^21^, ^22^ ^]^ (Figure S2). Such a fluoride content reduction lifts the symmetry in the number of electrons on the iron centers, and thus also the spin‐frustration. Indeed, FS‐DFT calculations on the reduced cluster coordinated with $N_{2}$ and two $H_{2}O$ molecules show that flipping the spin at the ${\mathrm{Fe}}^{\text{II}}$ center (green bar in Figure 3c) is no longer energetically favored over the ferromagnetic high‐spin configuration. Instead, only the two remaining ${\mathrm{Fe}}^{\mathrm{III}}$ centers can carry the β spin (Figure 3c), indicating the loss of spin‐frustration while the antiferromagnetic coupling between the ${\mathrm{Fe}}^{\mathrm{III}}$ centers is retained. This finding explains why, under the conventional assumption of a ferromagnetic high‐spin ground state for the [${\mathrm{Fe}}^{\text{II}}$ ${\mathrm{Fe}}_{2}^{\text{III}}(\mu_{3}$‐O)] node, Vitillo et al.^[^ ^48^ ^]^ had to artificially constrain the BDC linkers in their cluster model to prevent structural distortions that are not representative of the MOF crystal structure. FS‐DFT removes the need for any geometric constraint and yields a binding curve in line with the experimental findings by Li et al.^[^ ^15^ ^]^ (blue curve in Figure 4), namely a decreased N_2_‐binding affinity for the reduced cluster.

To investigate whether the change in binding affinity is caused by the loss of $F^{-}$ as a ligand or the partial reduction of the iron cluster, we calculated the dissociation curves for $N_{2}$ with the fully oxidized cluster (all three irons are ${\mathrm{Fe}}^{\mathrm{III}}$) without the counterion present (see Section S1.9). The solvation of the small anion is known to occur.^[^ ^70^ ^]^ We observe a somewhat stronger affinity for $N_{2}$ without $F^{-}$ present, the size of the change depends on whether the site that was bound to the anion remains undercoordinated or not. In either case, this clearly shows that it is the partial reduction of the Fe(III)–oxo cluster that reduces the $N_{2}$ binding affinity and not the absence of the counterion.

**Figure 5:**
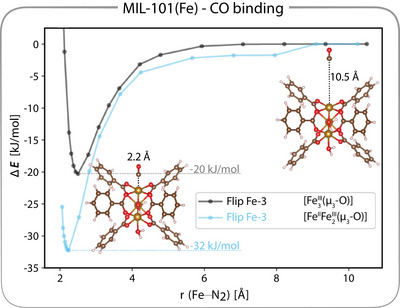
Energy profiles for CO binding, with unbound geometry (Fe─CO bond distance of 10.5 Å) set to 0 kJ/mol. The black curve corresponds to a configuration with spin flipped to β on the ${\mathrm{Fe}}^{\mathrm{III}}$–CO center for the [${\mathrm{Fe}}_{3}^{\text{III}}(\mu_{3}$‐O)] configuration. The blue curve represents flipping the spin of the water coordinated ${\mathrm{Fe}}^{\mathrm{III}}$ for the [${\mathrm{Fe}}^{\text{II}}$ ${\mathrm{Fe}}_{2}^{\text{III}}(\mu_{3}$‐O)] configuration. The energy gain upon CO coordination are given explicitly for each flip‐spin configuration.

Interestingly, CO binding has been reported to increase upon heating^[^ ^21^, ^22^ ^]^ opposite to the trend observed for $N_{2}$. Experiments were performed on MIL‐100(Fe), which consists of the same inorganic node as MIL‐101 but has benzene‐1,3,5‐tricarboxylate linkers instead. The change in CO coordination is attributed to the thermally induced reduction of the ${\mathrm{Fe}}^{\mathrm{III}}$ center with the additional negatively charged ligand (e.g., $F^{-}$). The additional d‐electron introduced upon reduction facilitates π‐back donation in addition to the σ donor interaction—a well‐known mechanism for stabilizing CO adsorbates.^[^ ^71^ ^]^ To probe this effect, we performed FS‐DFT dissociation energy scans for CO coordination to both the spin‐frustrated [${\mathrm{Fe}}_{3}^{\text{III}}(\mu_{3}$‐O)] cluster and its reduced form, [${\mathrm{Fe}}^{\text{II}}$ ${\mathrm{Fe}}_{2}^{\text{III}}(\mu_{3}$‐O)]. These calculations reveal a stronger binding interaction in the reduced cluster (blue curve in Figure 5) compared to the spin‐frustrated state (black curve), consistent with the experimental observation of increased CO affinity upon reduction at higher temperatures (see also Section S1.9).^[^ ^21^, ^22^ ^]^ This theoretical insight supports the interpretation that the added d‐electron enhances CO stabilization through π‐back donation and may also be associated with the observed loss of spin frustration in the reduced cluster.

## Conclusion

Our work demonstrates that the widely used standard DFT description with M=16 of MIL‐101(Fe) is fundamentally flawed, even at a qualitative level. The ferromagnetic approximation fails to reproduce the correct geometry of the electronic ground state, resulting instead in significant structural distortions and thus incorrect predictions of the stability of the MOF. In contrast, by explicitly accounting for competing spin alignments using flip‐spin DFT for a finite cluster model of the MIL‐101(Fe), the correct linker orientation is retained. We also showed that spin‐frustration is not an artifact of finite models but persists in the extended lattice of MIL‐88B(Fe).

Furthermore, we demonstrate how spin‐frustration governs the temperature‐dependent adsorption behavior of small molecules in these MOFs. The experimentally observed decline of $N_{2}$ fixation activity and increase in CO binding upon thermal reduction of one ${\mathrm{Fe}}^{\mathrm{III}}$ center can only be explained, when accounting for the antiferromagnetic coupling of the iron centers.

Taken together, our findings establish magnetic frustration as an intrinsic feature of iron‐based MOFs with triangular motifs, underscoring the necessity of including it into any reliable theoretical treatment. Ferromagnetic, high‐spin DFT approaches, despite their prevalence, yield not only quantitatively incorrect but also qualitatively misleading insights into such systems.

## Conflict of Interests

The authors declare no conflict of interest.

## Supporting information

Supporting Information

## Data Availability

Details on the computational methods can be found in the supplementary information document, which contains i) a description of how the cluster was cut from the crystal structure, ii) details on the geometry scans, iii) a comparison of how density functionals from different rungs of Jacob's ladder affect the energy difference between ferromagnetic and spin‐frustrated configuration, and iv) a detailed description of the periodic calculations and their results. To enable reproduction of our work, the input files and structures for the cluster and periodic calculations can be found in the Zenodo archive https://doi.org/10.5281/zenodo.16569889.
