# Supplementary material for: Spin Frustration Determines the Stability and Reactivity of Metal–Organic Frameworks with Triangular Iron(III)–Oxo Clusters
Source: Angew Chem Int Ed Engl. 2025 Sep 9;64(41):e202514014. doi: 10.1002/anie.202514014 (PMC12501687; doi:10.1002/anie.202514014)
Supplement: Supplementary file 1 — Supporting Information [file ANIE-64-e202514014-s001.pdf]

Supporting Information:

Spin-Frustration Determines the Stability and Reactivity of Metal-Organic Frameworks with Triangular Iron(III)-oxo Clusters

Patrick Lechner,[a,d] Gaurab Ganguly,[a] Michael J. Sahre,[b] Georg Kresse,[b,c] Johannes C. B. Dietschreit,*[a] Leticia González*[a]

[a] P. Lechner, Dr. G. Ganguly, Dr. J. C. B. Dietschreit, Prof. L. González**
Institute of Theoretical Chemistry, Faculty of Chemistry, University of Vienna, Währinger Str. 17, 1090 Vienna, Austria

[b] Dr. M. J. Sahre, Prof. G. Kresse
University of Vienna, Faculty of Physics, Kolingasse 14-16, A-1090 Vienna, Austria

[c] VASP Software GmbH, Berggasse 21/14, A-1090 Vienna, Austria

[d] University of Vienna, Vienna Doctoral School in Chemistry (DoSChem), Währinger Str. 42, 1090 Vienna, Austria.

E-mail: leticia.gonzalez@univie.ac.at;johannes.dietschreit@univie.ac.at

Table of Contents of the Supporting Information:

S1 Density functional (DFT), Broken-symmetry DFT (BS-DFT), and Flip-Spin DFT (FS-DFT) calculations S-3

S1.1 Computational Details S-3

S1.2 Cluster cut-out S-3

S1.3 Geometry scans for N_2 and CO binding S-3

S1.4 Jacobs ladder for FS-DFT S-4

S1.5 Example Input BS & FS-DFT S-5

S1.6 Add on Figure 2E S-7

S1.7 $[Fe^{II}Fe_2^{III}(\mu_3{-}O)]$ S-7

S1.8 Failed N_2 binding curves with standard DFT S-8

S1.9 Loss of Anion S-9

S1.10 Energy difference antiferromagnetic HS vs. ferromagnetic HS after CO binding S-10

S2 Structure of MIL-101(Fe) vs MIL-88B(Fe) S-11

S3 Periodic-DFT calculations on MIL-88B(Fe) S-12

S1 Density functional (DFT), Broken-symmetry DFT (BS-DFT), and Flip-Spin DFT (FS-DFT) calculations

S1.1 Computational Details

For the cluster model, all calculations – UKS-DFT, Broken-symmetry DFT (BS-DFT)[S1], and Flip-Spin DFT (FS-DFT)[S1,S2] – were carried out with the ORCA code version 6.0.1[S3]. Unless state otherwise, the cluster was described at the PBE0-D3(BJ)/def2-SVP[S4–S7] level of theory, where we used the def2-TZVP basis for the Fe atoms. If not modeled explicitly, interactions with the water molecules present in this MOF were described implicitly by using the conductor-like polarizable continuum model (CPCM)[S8]. To increase computational efficiency for geometry optimization calculations, we used the RIJCOSX[S9] approximation. To account for spin contamination in the BS-DFT and the Flip-Spin method, the energies were corrected with the approximate spin projection (AP) method by Yamaguchi.[S10] The FS-DFT was performed, by using the keywords `FlipSpin` and `FinalMS` in the `scf` block.

S1.2 Cluster cut-out

The initial geometry was obtained by cutting out a cluster from the crystal structure of MIL-101(Fe) (crystallography open database (COD) entry number 4000663[S11]). The free carboxylate moieties of the BDC linkers were replaced with hydrogen atoms to obtain a charge-neutral cluster. The resulting cluster was used as a guess structure for a geometry optimization in the ferromagnetic high spin (M=16) state. To speed up the initial unrestricted DFT, BS-DFT, and FS-DFT calculations, the cluster model was further truncated by replacing the benzene rings with CH_3 groups effectively turning benzoic into acetic acid. This truncation was justified, because the benzol rings of the BDC linkers did not affect the electronic structure of the irons. The structure with the acetic acid ligands, optimized in the $M = 16$ state, was used as input structure for the Jacob's ladder calculations, the comparison of unrestricted and broken-symmetry DFT, and investigation of Flip-Spin DFT. All calculations regarding N_2 and CO binding as well as coordination with explicit water molecules were performed with the full cluster mode (*i.e.*, using benzoic acid ligands).

S1.3 Geometry scans for N_2 and CO binding

For calculations using the benzoic acid cluster, the irons of the ferromagnetic high spin optimized cluster model were coordinated with the corresponding ligands (H_2O, and N_2, or CO). Since high spin DFT geometry optimization failed for the coordinated cluster model (see Figure 3A), the FS-DFT optimized cluster model was used as input for calculating the geometry scans shown in Figure 4 and 5. It is important to note that while the initial structure optimization was performed using the setup described above, the D3(BJ) dispersion correction had to

be excluded during the subsequent geometry scans to avoid unphysical structural distortions (the BDC linkers are not restrained by the crystal nor are the interactions between dissociating ligands and BDC linkers screened by explicit solvent molecules, which leads to an overly strong D3 interaction between BDC linker and dissociating small molecule ligand). To investigate the N_2|CO bonding, geometry scans in the Flip-Spin approach were carried out, starting from the FS-DFT optimized structure and elongating or shortening the Fe$-$N$_2$ | Fe$-$N$_2$ bond.

S1.4 Jacobs ladder for FS-DFT

In order to compare different levels of DFT, we performed calculations with functionals from different rungs of the DFT Jacob's ladder. The calculations were performed with the Flip-Spin method and the following functionals: PBE[S12], revTPSS[S13], R^{2}SCAN[S14], PBE0[S4,S5], MO6-L[S15], MO6-2X[S16], ωB97M-V[S17], and the double hybrid functional B2-PLYP.[S18] For the B2-PLYP functional, the MP2 correlation part was accelerated with the def2-SVP/C[S19] auxiliary basis set.

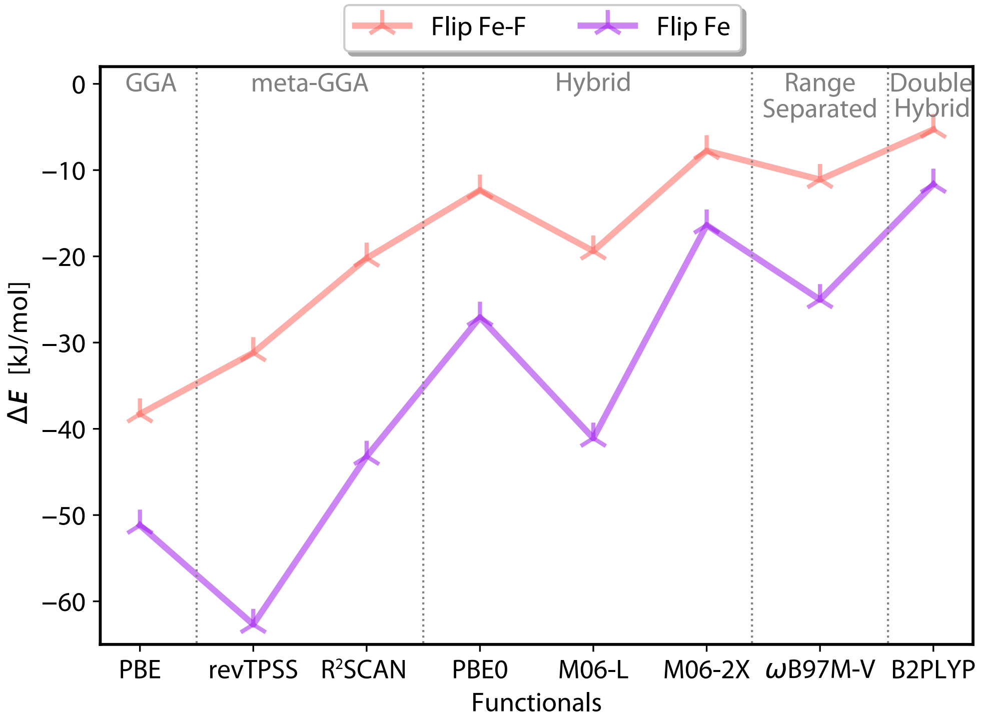

Figure S1: Trend along the DFT Jacob's ladder of the energy difference (ΔE) between high-spin and low spin configuration for flipping spins on either Fe-F or an Fe with no additional ligands. Dashed vertical gray lines group functionals into different rungs of the Jacob's ladder.

As Figure S1 shows, the energy gap between the spin-frustrated and ferromagnetic solution is largest for GGA and meta-GGA functionals, whereas double-hybrid functionals yield a significantly smaller gap. This trend is consistent with the work of Reiher,[S20–S22] who demon-

strated that GGA functionals preferentially stabilize states with low total spin, while hybrid functionals are prone to artificially stabilize the ferromagnetic high-spin configurations in iron complexes. Hybrid functionals like PEB0 present a reasonable middle ground. Regardless, all functionals converge on the spin-frustrated configuration as the energetically preferred state.

The geometry used for the comparison of different functionals was obtained from a FS-DFT optimization with PBE0.

S1.5 Example Input BS & FS-DFT

The following example ORCA inputs were used for the BS-DFT and FS-DFT ($M = 6$) geometry optimizations of MIL-101(Fe).

```
ORCA Input File for BS-DFT Geometry Optimization (M = 6)
-----------------------------------------------

! def2-SVP PBE0 D3BJ CPCM(Water) TightSCF defgrid3 SlowConv soscf notrah
   ↪ opt RIJCOSX

%basis
newgto Fe "def2-TZVP" end
end

%scf
  BrokenSym 10,5      # number α, and β spins
  sthresh 9e-6
end

* xyzfile 0 16 MIL-101_BS.xyz  # Multiplicity must be set to high spin (M
   ↪ = 16)
```

```
ORCA Input File for FS-DFT (Final MS = 5/2 (M = 6))
--------------------------------------------

! def2-SVP PBE0 D3BJ CPCM(Water) TightSCF defgrid3 SlowConv soscf notrah
    ↪ opt RIJCOSX

%basis
newgto Fe "def2-TZVP" end
end

%scf
  Flipspin 0      # Index of Atom(s) on which α flipped to β spins
  FinalMS 2.5     # needs to be set for the desired multiplicity for M = 6
      ↪         # Final MS = 5/2
  sthresh 9e-6
end

%geom
  ReducePrint false # Full output to check for desired multiplicity (M)
end

* xyzfile 0 16 MIL-101_BS.xyz   # Multiplicity must be set to high spin (M
    ↪  = 16)
```

S1.6 Add on Figure 2E

As shown in Table S1, extending the small cluster model by including the BDC linker does not affect the energy gap between the ferromagnetic ($M = 16$) and spin-frustrated configurations (see also Figure 2E).

Table S1: Results of FS-DFT by using the cluster model with BDC linkers

Cluster with BDC linker	
**Flipped spin on #Fe**	**ΔE [kJ/mol]**
1	-13.28
2	-29.39
3	-29.39

S1.7 $[Fe^{II}Fe_2^{III}(\mu_3{-}O)]$

Figure S2 illustrates the resulting electronic configuration following the thermal reduction (T > 100 °C) of one Fe^{III} center, induced by fluoride release.

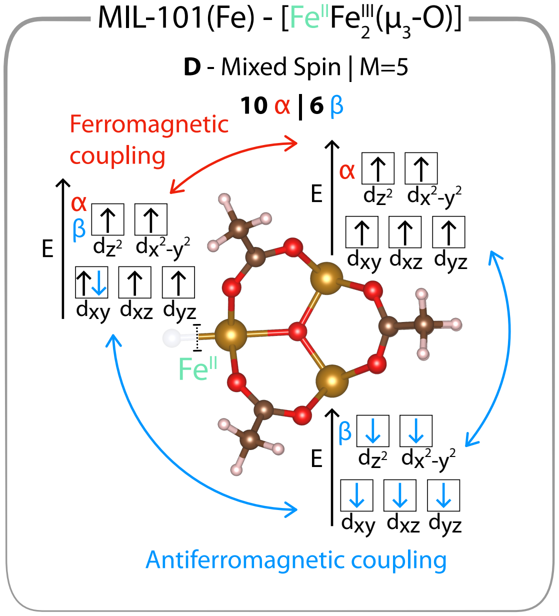

Figure S2: Electron configuration of MIL-101(Fe) upon heating and reduction of one Fe^{III} to Fe^{II}

S1.8 Failed N_2 binding curves with standard DFT

Figure S3 displays the N_2 binding energy profiles computed using standard DFT in the ferromagnetic high-spin configuration. The corresponding structure highlights the severe distortion that arises during geometry scans performed with this method, underscoring its failure to capture the correct binding behavior and predicting stable structures.

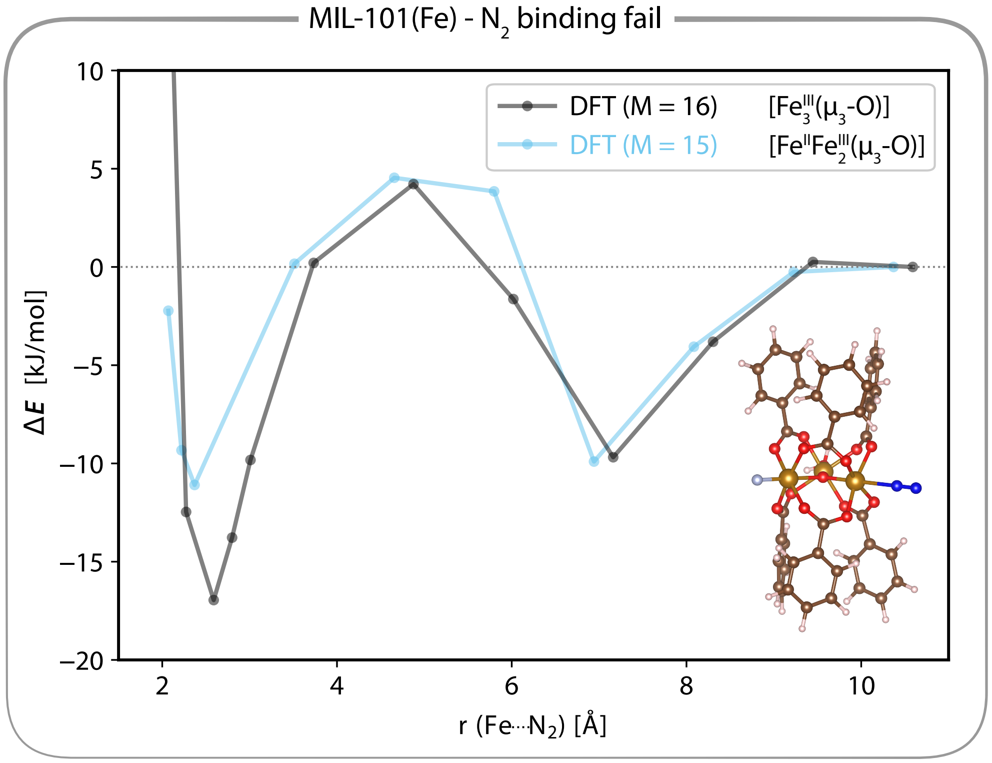

Figure S3: Failed N_2 binding curves, calculated with standard DFT, for the ferromagnetic high spin cases. The depicted structure shows the distorted structure, resulting from geometry scans carried out with standard DFT.

S1.9 Loss of Anion

To analyze whether the change in the binding affinity of the Fe(III)-oxo cluster is caused by the loss of the anion (in our case F^-) or the partial reduction of one of the three irons, we performed simulations with the cluster model without the anion, but with all three Fe atoms in oxidation state III. This cluster has a single positive charge. Exposed to a polar solvent, the solvation of the singly charged counterion is known to occur.[S23]

Without any water ligands, the three iron atoms in the cluster model are now identical, giving rise to "perfect" spin frustration (see Figure S4A). As one might expect, the spin-frustrated state is now even slightly more stable than the ferromagnetic high-spin configuration (35 kJ/-mol without F^- and 30 kJ/mol with counter ion). The coordination by any ligand, charged or not, will break this symmetry.

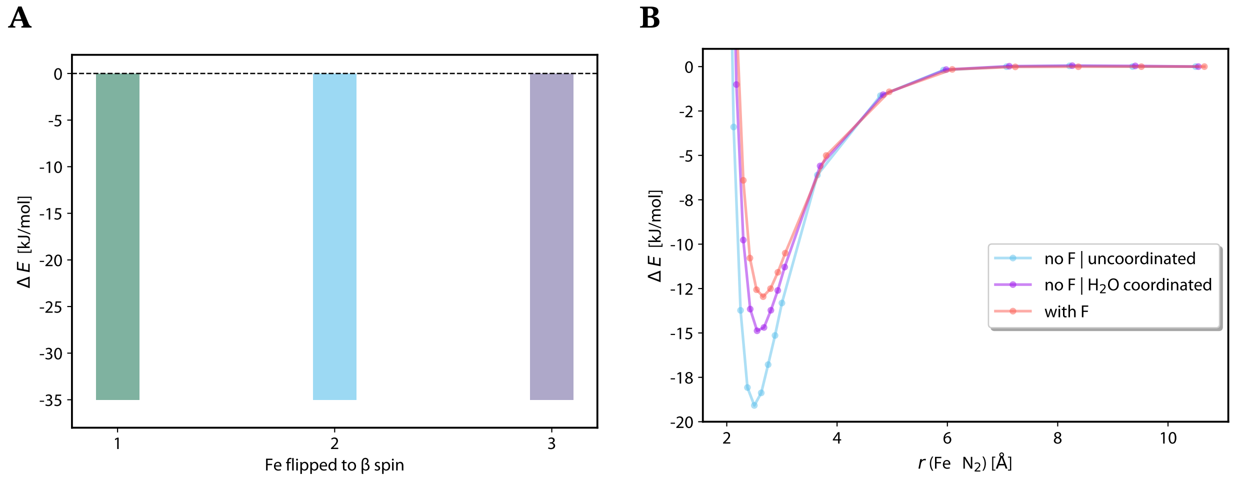

Figure S4: Simulations of the cluster model without negative counter ion. **A**, energy difference between ferromagnetic and spin-frustrated high-spin states for β-spins localized at any of the three Fe(III) atoms. **B**, flip-spin dissociation curve for N_2 from the positively charged cluster with and without the presence of an additional water ligand at the freed-up iron-site.

To test the influence of the presence of the counterion on the binding affinity, we calculated dissociation curves for N_2 from the positively charged cluster (no Fe atom is reduced), the results are shown in Figure S4B. N_2 binds more strongly to the cluster without counterion than when F^- is present. The presence of a water ligand on the Fe atom that no longer binds fluoride modulates this stabilization. With one Fe under coordinated the stabilization is 8 kJ/-mol, whereas with both other irons binding water the stabilization drops to only 3 kJ/mol, likely because the highly polar ligands lift the symmetry between the Fe atoms. Hence, we can conclude that it is not the loss of the anion that changes the binding affinity of the Fe-oxo cluster but the partial reduction.

S1.10 Energy difference antiferromagnetic HS vs. ferromagnetic HS after CO binding

When the complex is coordinated with both H_2O and CO (Figure S5) at low temperatures (T<100°), the spin-frustrated configuration is also predicted to be most favorable, giving an energy difference with the high spin situation ($M = 16$).

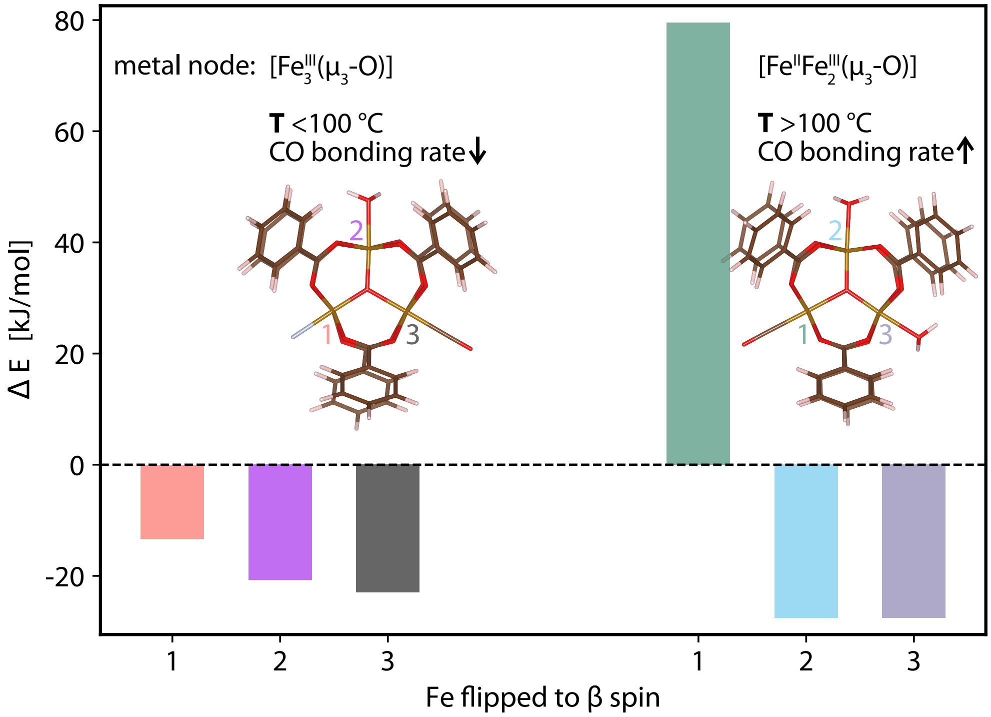

Figure S5: Energy difference (ΔE) between FS-DFT calculated clusters and the ferromagnetic high-spin ($M = 16$) reference. Each bar represents the energy of flipping the spin to β down on the corresponding Fe^{III} or Fe^{II}.

FS-DFT calculations on the reduced cluster (see also Figure S2) coordinated with CO and two H_2O molecules show that flipping the spin at the Fe^{II} center (green bar in Figure S5) is no longer energetically favored over the ferromagnetic high-spin configuration. Instead, only the two remaining Fe^{III} centers can carry the β spin (blue and violett bar Figure S5), indicating the loss of spin-frustration while antiferromagnetic coupling is retained.

S2 Structure of MIL-101(Fe) vs MIL-88B(Fe)

Figure S6 shows that MIL-101 and MIL-88B share the same [$Fe_3^{III}(\mu_3{-}O)$] nodes and BDC linkers, but differ in their overall crystal structures, leading to distinct pore sizes.

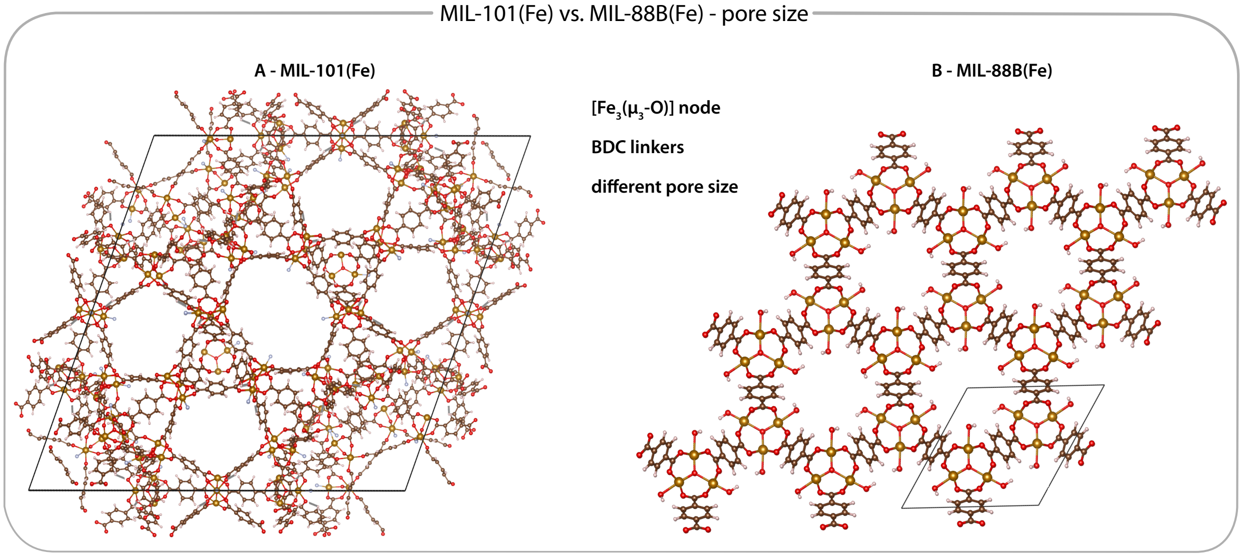

Figure S6: Pore size comparison of the crystal structures of MIL-101(Fe) and MIL-88B(Fe)

S3 Periodic-DFT calculations on MIL-88B(Fe)

The periodic calculations are based on the experimental crystal structure of MIL-88B(Fe), which is listed in the Cambridge Crystallographic Data Center (CCDC) under the number 2088535. This structure contains dangling oxygen atoms at the iron sites. To better compare with the cluster model, one oxygen was replaced with fluorine, and the other two were removed. This resulted in the structure displayed in Figure S7A. Different spin states were obtained by setting the initial magnetic moments of the Fe atoms accordingly. For example, an initial spin state in which the α-electrons are localized at Fe-1 and Fe-2 and the β-electrons are localized at Fe-3 can be achieved by setting the magnetic moments of the Fe atoms to 5, 5, and -5 μ_B, respectively. We confirmed that these initial spin states did not change during electronic relaxation by inspecting the total magnetic moment of the unit cell and the local magnetic moments of the Fe atoms after optimizing the electronic structure.

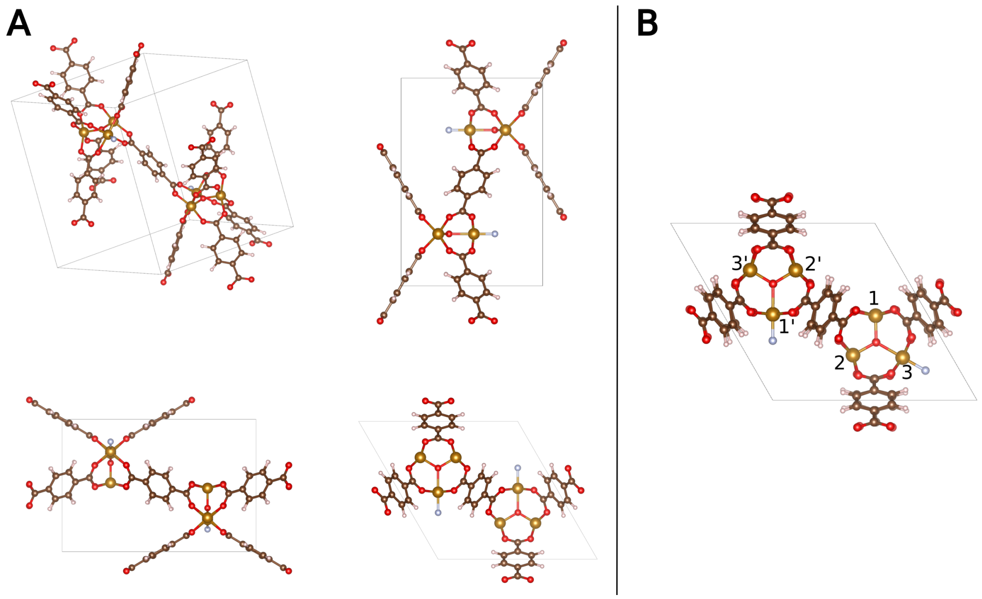

Figure S7: Panel A: The unit cell of MIL-88B(Fe) viewed from four different angles. One iron ion in each of the two metal clusters is coordinated by a F^--ion. Panel B: The unit cell with an alternative coordination pattern for the fluoride ligands.

The geometry of the modified structure in Figure S7A was relaxed for each spin state until the forces on the nuclei were below 0.02 eV/Å using the experimental lattice parameters, *i.e.* only the positions of the nuclei were optimized. The structure in Figure S7B was relaxed for the high spin state and the spin-frustrated state in which the β-electrons are localized at the Fe-sites 2 and 2'. The structure optimization was carried out with the spin-polarized (meaning unrestricted) PBE0 functional and a cutoff of 400 eV at the Γ-point using the VASP code.[S24]

We checked the energy convergence with respect to the plane wave cutoff by performing

single-point calculations for the different spin states at their optimal geometry with a plane wave cutoff of 520 eV. The energy per unit cell of the spin states relative to the ferromagnetic state changed by 5 meV or less as the plane wave cutoff increased (see Table S2).

Table S2: Comparison of energy differences per unit cell of different spin states relative to the ferromagnetic state at a plane wave cutoff of 400 eV and 520 eV, with the difference in differences $\Delta E_{400} - \Delta E_{520}$. The iron site indices at which the β-electrons are localized follow the nomenclature in Figure S8B. All energies are in eV.

Sites with β-electrons	ΔE_{400}	ΔE_{520}	$\Delta E_{400} - \Delta E_{520}$
11’	-0.251	-0.248	-0.003
12’	-0.466	-0.469	0.002
13’	-0.462	-0.465	0.002
22’	-0.661	-0.662	0.002
23’	-0.661	-0.663	0.002
33’	-0.662	-0.667	0.005

The energy convergence with respect to the k-point mesh was checked by repeating calculations for the optimized structures with a 2x2x2 k-point mesh. Similar to the increased plane wave cutoff, energy differences with respect to the ferromagnetic state changed only by 4 meV or less when the number of k-points was increased (see Table S3).

Table S3: Comparison of energy differences per unit cell of different spin states relative to the ferromagnetic state for Γ-point only and a 2x2x2 k-point mesh, with the difference in differences $\Delta E_{\Gamma} - \Delta E_{222}$. The iron site indices at which the β-electrons are localized follow the nomenclature in Figure S8B. All energies are in eV.

Sites with β-electrons	ΔE_{Γ}	ΔE_{222}	$\Delta E_{\Gamma} - \Delta E_{222}$
11’	-0.251	-0.249	-0.001
12’	-0.466	-0.462	-0.004
13’	-0.462	-0.459	-0.003
22’	-0.661	-0.657	-0.004
23’	-0.661	-0.657	-0.004
33’	-0.662	-0.658	-0.004

Non-collinear calculations were performed using the optimized electronic ground state structure in which the β-electrons are localized at Fe-3 and Fe-3’ (see Figure S8). We employed the LDA functional with a Hubbard correction of U = 4 eV for the Fe atoms using the DFT+U approach by Dudarev *et al.*[S25] Initial magnetic moments were set to yield either a zero net moment (Figure S9A, state 1) or a magnetic moment of $5\mu_B$ (Figure S9C, state 2). Convergence with respect to the plane-wave cutoff and the k-point mesh was verified by repeating

the calculations for states 1 and 2 with a cutoff of 520 eV and a 2x2x2 k-point mesh. After electronic structure optimization, the relative energy between states 1 and 2 changed by less than 0.06 meV when increasing either the cutoff or the number of k-points.

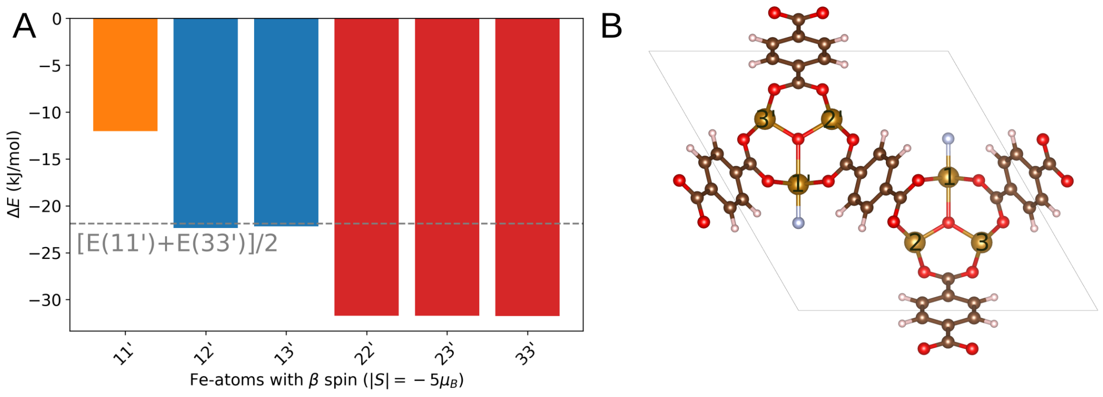

Figure S8: Energy differences (ΔE) of various spin-frustrated configurations in MIL-88B relative to the high-spin reference state (panel A). Each configuration is labeled by the site indices where β-spin electrons are localized (see panel B for site labeling). The energies are normalized by dividing the energy of the cell by two for better comparison to the cluster calculations.

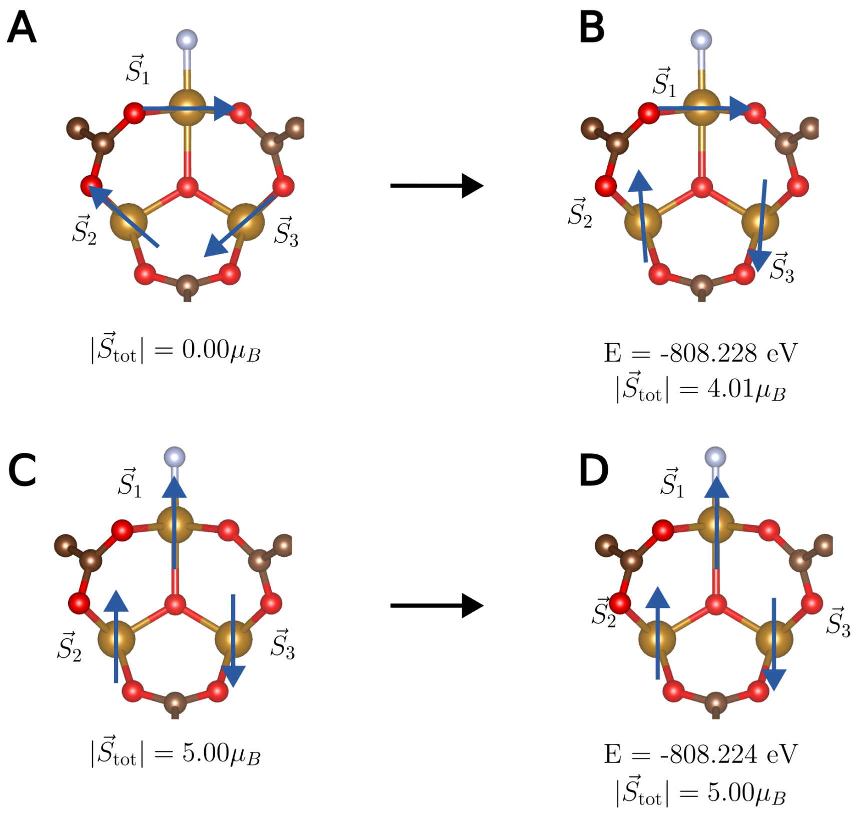

Figure S9: The local spin configuration (see also Table S4) at the Fe-sites in the electronic ground state of MIL-88B(Fe) obtained from non-collinear DFT (LDA+U). Starting with the antiferromagnetic configuration as initial guess for the spin orientation (Panel A), the spin configuration changes to the spin-frustrated one after electronic structure relaxation (Panel B). If initialized as spin-frustrated (Panel C), the system remains in this spin state after relaxation (Panel D).

Table S4: Localized spin (in μ_B) at the Fe-sites from projection of the spin density onto localized basis functions[S26] after non-collinear electronic structure relaxation with initial magnetic moments as in Figure S9A to the configuration Figure S9B. Values are averaged over the two identical Fe-sites in the two Fe-cluster per unit cell.

	Fe-1	Fe-2	Fe-3
S_x	4.08	-0.36	-0.41
S_y	0.24	3.93	-3.92
S_z	0.00	0.01	-0.01

References

(S1) Noodleman, L. *J. Chem. Phys.* **1981**, *74*, 5737–5743.

(S2) Neese, F. *J. Phys. Chem. Solids* **2004**, *65*, 781–785.

(S3) Neese, F. *WIREs Comput. Mol. Sci.* **2022**, *12*, e1606.

(S4) Perdew, J. P. *Phys. Rev. B* **1986**, *33*, 8822–8824.

(S5) Adamo, C.; Barone, V. *J. Chem. Phys.* **1999**, *110*, 6158–6170.

(S6) Weigend, F.; Ahlrichs, R. *Phys. Chem. Chem. Phys.* **2005**, *7*, 3297.

(S7) Grimme, S.; Ehrlich, S.; Goerigk, L. *J. Comput. Chem.* **2011**, *32*, 1456–1465.

(S8) Cossi, M.; Rega, N.; Scalmani, G.; Barone, V. *J. Comput. Chem.* **2003**, *24*, 669–681.

(S9) Neese, F.; Wennmohs, F.; Hansen, A.; Becker, U. *Chem. Phys.* **2009**, *356*, 98–109.

(S10) Yamaguchi, K.; Jensen, F.; Dorigo, A.; Houk, K. *Chem. Phys. Lett.* **1988**, *149*, 537–542.

(S11) Lebedev, O. I.; Millange, F.; Serre, C.; Van Tendeloo, G.; Férey, G. *Chem. Mater.* **2005**, *17*, 6525–6527.

(S12) Perdew, J. P.; Ernzerhof, M.; Burke, K. *J. Chem. Phys.* **1996**, *105*, 9982–9985.

(S13) Perdew, J. P.; Ruzsinszky, A.; Csonka, G. I.; Constantin, L. A.; Sun, J. *Phys. Rev. Lett.* **2009**, *103*, 026403.

(S14) Furness, J. W.; Kaplan, A. D.; Ning, J.; Perdew, J. P.; Sun, J. *J. Phys. Chem. Lett.* **2020**, *11*, 8208–8215.

(S15) Zhao, Y.; Truhlar, D. G. *J. Chem. Phys.* **2006**, *125*, 194101.

(S16) Zhao, Y.; Truhlar, D. G. *Theor. Chem. Acc.* **2008**, *120*, 215–241.

(S17) Mardirossian, N.; Head-Gordon, M. *J. Chem. Phys.* **2016**, *144*, 214110.

(S18) Grimme, S. *J. Chem. Phys.* **2006**, *124*, 034108.

(S19) Weigend, F. *Phys. Chem. Chem. Phys.* **2006**, *8*, 1057.

(S20) Reiher, M. *Inorg. Chem.* **2002**, *41*, 6928–6935.

(S21) Swart, M.; Gruden, M. *Acc. Chem. Res.* **2016**, *49*, 2690–2697.

(S22) Rosen, A. S.; Notestein, J. M.; Snurr, R. Q. *ACS Catal.* **2019**, *9*, 3576–3587.

(S23) Siwaipram, S.; Bopp, P. A.; Keupp, J.; Pukdeejorhor, L.; Soetens, J.-C.; Bureekaew, S.; Schmid, R. *The Journal of Physical Chemistry C* **2021**, *125*, 12837–12847.

(S24) VASP Software GmbH; Vienna Ab initio Simulation Package. 2025; `https://www.vasp.at/`, Accessed: 2025-05-26.

(S25) Dudarev, S. L.; Botton, G. A.; Savrasov, S. Y.; Humphreys, C. J.; Sutton, A. P. *Phys. Rev. B* **1998**, *57*, 1505–1509.

(S26) Schüler, M.; Peil, O. E.; Kraberger, G. J.; Pordzik, R.; Marsman, M.; Kresse, G.; Wehling, T. O.; Aichhorn, M. *J. Phys. Condens. Matter* **2018**, *30*, 475901.
